# GPR143, a novel immunohistochemical marker for renal tumors with *FLCN/TSC/MTOR-TFE* alterations

**DOI:** 10.1007/s00428-026-04646-4

**Published:** 2026-07-17

**Authors:** Qinyuan Li, Anupama Singh, Rong Hu, Wei Huang, Daniel D. Shapiro, E. Jason Abel, Yang Zong

**Affiliations:** 1 Department of Pathology and Laboratory Medicine, School of Medicine and Public Health, University of Wisconsin-Madison, 600 Highland Avenue, Madison, WI 53792 USA; 2 Department of Urology, School of Medicine and Public Health, University of Wisconsin-Madison, Madison, WI USA

**Keywords:** Renal cell carcinoma, TFE, Translocation, GPR143, GPNMB

## Abstract

Although several ancillary tests are available in limited laboratories, diagnosis of microphthalmia (MiT)/TFE family translocation renal cell carcinoma (tRCC) could be challenging due to diverse and overlapping tumor morphology and the lack of reliable biomarkers. GPNMB has been recently identified as a diagnostic marker for various renal neoplasms with *FLCN/TSC/mTOR-TFE* alterations. However, the sensitivity and specificity of GPNMB immunostain are suboptimal and the result interpretation in ambiguous cases could be difficult. To search additional biomarkers that could improve the screening sensitivity and predict genetic aberrations in *FLCN/TSC/mTOR-TFE* pathway in renal tumors, we performed bioinformatic analysis of publicly available cancer databases and found GPR143, a transmembrane protein regulated by MiT transcription factors, was highly expressed in a subset of renal cell carcinomas (RCCs). In two kidney cancer cohorts of the Cancer Genome Atlas (TCGA), RCCs with high levels of *GPR143* expression were enriched for renal neoplasms with *FLCN/TSC/mTOR-TFE* alterations. Similar to GPNMB labeling, GPR143 immunostain was positive in the majority of tRCC cases and renal tumors with *FLCN/TSC/mTOR* alterations, suggesting that GPR143 could function as another surrogate marker for *FLCN*/*TSC/mTOR-TFE* alterations in certain renal tumors. Interestingly, despite the concordant GPR143 and GPNMB immunoreactivity in most renal neoplasms with *FLCN/TSC/mTOR-TFE* alterations, diffuse GPR143 immunostain was observed in some cases with negative or focal GPNMB labeling. Taken together, our results indicate GPR143 could serve as a useful adjunct marker to improve the sensitivity for screening renal tumors with *FLCN/TSC/mTOR-TFE* alterations.

**Supplementary Information:**

The online version contains supplementary material available at https://doi.org/10.1007/s00428-026-04646-4.

## Introduction

GPNMB (Glycoprotein nonmetastatic melanoma protein B), a transmembrane protein regulated by the microphthalmia (MiT)/TFE family of transcription factors, has been recently discovered as a diagnostic biomarker for various renal neoplasms with *FLCN/TSC/mTOR-TFE* alterations [1, 2]. High expression of GPNMB was found in MiT/TFE family translocation renal cell carcinoma (tRCC) [1–3], renal tumors associated with Birt–Hogg–Dubé (BHD) syndrome or somatic *FLCN* mutations [4–6], and many renal tumors associated with *TSC/mTOR* signal pathway aberrations, including renal cell carcinoma (RCC) with fibromyomatous stroma, [7–9] eosinophilic solid and cystic renal cell carcinoma (ESC RCC), low grade oncocytic tumor (LOT) and eosinophilic vacuolated tumor (EVT) [1, 2, 6] as well as angiomyolipoma [1, 2]. However, approximately 13%—30% of tRCC cases are negative for diffuse GPNMB staining [1, 2]. Furthermore, a large fraction of chromophobe renal cell carcinoma (chRCC) show diffuse or partial positive immunoreactivity with GPNMB antibody [1, 2, 6]. Several other renal epithelial tumors including oncocytoma, clear cell renal cell carcinoma (ccRCC) and papillary renal cell carcinoma (pRCC) could rarely exhibit diffuse or focal staining for GPNMB [2, 5, 6].

Given that many renal neoplasms with *FLCN/TSC/mTOR-TFE* alterations also express several downstream targets of the MiT/TFE family of transcription factors, such as Melan-A, HMB45, cathepsin K and TRIM63 [10–13], we postulated that immunohistochemistry (IHC) analysis with antibodies against additional MiT/TFE downstream targets could provide useful adjunct tests to existing markers and may improve the sensitivity and specificity for the diagnosis of renal tumors with *FLCN/TSC/mTOR-TFE* alterations.

GPR143 protein is a unique G protein-coupled transmembrane receptor protein involved in the pathogenesis of ocular albinism [14]. It has been identified as a downstream target of MiT transcription factors, which is mainly localized in intracellular organelles, such as late endosomes and melanosomes, in pigment cells [15–17]. In addition to its important role in melanosome biogenesis and maturation, it has recently reported that GPR143 is also implicated in exosome biogenesis and promotes cell mobility and cancer metastasis [18]. In a genetically engineered mouse model of *TFE3*-rearranged RCC, both GPNMB and GPR143 were significantly overexpressed in mouse kidneys [19]. In this study, we found that in the Cancer Genome Atlas (TCGA) kidney cancer cohorts, human renal cell carcinomas (RCCs) with high levels of GPR143 expression were enriched for renal neoplasms with *FLCN/TSC/mTOR-TFE* alterations. Similar to GPNMB, IHC analysis with an antibody against GPR143 was positive in the majority of tRCC cases and renal tumors with *FLCN/TSC/mTOR* alterations, suggesting that GPR143 could function as another surrogate marker for *FLCN* mutations and *TSC/mTOR-TFE* signal pathway aberrations in certain renal tumors.

## Materials and methods

### Bioinformatics tool and data analysis

To examine the expression of GPR143 in RCCs, we performed in silico data analysis using the following open-access cancer research databases and interactive web portals: 1. The Human Protein Atlas (HPA) database (http://www.proteinatlas.org), which is the most comprehensive database for evaluating the distribution of all the human proteins in normal cells, tissues and organs as well as major cancer types [20]. The mRNA data of GPR143 expression from 21 cancer types and 3 major RCC subtypes were obtained through the HPA database. 2. cBioPortal database (https://www.cbioportal.org) is a web-based public platform for interactively exploring multidimensional cancer genomics datasets [21]. Specific gene fusions, gene mutations, amplifications or other genetic changes of the *FLCN/TSC/mTOR-TFE* pathway in RCCs were identified by manual inquiry in TCGA datasets (TCGA-KIRC, TCGA-KIRP and TCGA-KICH cohorts) via cBioPortal.

### Case selection

We retrospectively reviewed the electronic medical record from our surgical pathology laboratory registry in the University of Wisconsin Hospital and Clinics and identified 27 renal tumors closely associated with *FLCN/TSC/mTOR-TFE* alterations from 26 patients between 2005 through May 2026. The cohort included ten tRCC cases (eight *TFE3*-rearranged RCC either diagnosed by histology with unequivocal TFE3 IHC test (*n* = 2) or confirmed by *TFE3* fluorescence in-situ hybridization (FISH) analysis (*n* = 6), one case of FISH confirmed *TFEB*-rearranged RCC with *MALAT1::TFEB* fusion and one case of *TFEB*-amplified RCC confirmed by next generation sequencing), four *FLCN* mutated renal tumors (two hybrid oncocytic chromophobe tumors (HOCTs), one unclassified low grade oncocytic renal neoplasm and one ccRCC) from three BHD syndrome patients diagnosed with relevant clinical history and positive germline *FLCN* mutations, two *TSC1*-mutated RCCs from two patients with tuberous sclerosis complex (TSC), one ESC RCC case with a germline *TSC1* mutation, two LOTs, two EVTs, and six cases of renal angiomyolipoma (AML) including a patient with clinical TSC syndrome and a patient with a germline *TSC2* deletion (Table 1). This study was approved by the Institutional Review Board of School of Medicine and Public Health, University of Wisconsin-Madison.

**Table 1:** Clinicopathological characteristics of 27 renal tumors from 26 patients

Case	Sex/age	Diagnosis	Hereditary cancer syndrome	Germline testing	FISH analysis	TFE3 IHC	GPNMB IHC	GPR143 IHC
1	F/5	*TFE3-*rearranged RCC	N/A	N/A	ND	+	DP	DP
2	F/55	*TFE3-*rearranged RCC	N/A	N/A	ND	+	DP	DP
3	M/44	*TFE3-*rearranged RCC	N/A	N/A	+	+	DP	DP
4	M/47	*TFE3-*rearranged RCC	N/A	N/A	+	+	FP	DP
5	F/64	*TFE3-*rearranged RCC (Biphasic)	N/A	N/A	+	+	FP	DP
6	F/42	*TFE3-*rearranged RCC	N/A	N/A	+	+	Neg	Neg
7	F/19	*TFE3-*rearranged RCC	N/A	No pathogenic mutations	+	+	DP	DP
8	F/43	*TFE3-*rearranged RCC	N/A	N/A	+	+	DP	DP
9	F/16	*TFEB-*rearranged RCC	N/A	N/A	+	ND	DP	DP
10	M/85	*TFEB-*amplified RCC	N/A	N/A	ND	ND	FP	Neg
11	F/39	HOCT (n = 2)	BHD	*FLCN c.1286dupA* (p.H429Qfs*27)	ND	ND	DP	DP
12	M/79	Low grade oncocytic neoplasm	BHD	*FLCN (c.1285dupC)* (p.H429Pfs*27)	ND	ND	DP	DP
13	M/55	Clear cell RCC	BHD	*FLCN c.1285dup* (p.His429fs)	ND	Neg	Neg	Neg
14	F/26	ESC RCC	N/A	*TSC1 (c.1218C* > *A)* (p.Y406*)	ND	Neg	DP	DP
15	F/38	*TSC1-*mutated RCC	TSC	*TSC1 (c.364-2A* > *G)*	ND	Neg	DP	Neg
16	F/33	*TSC1-*mutated RCC	TSC	*TSC1 (c.648–649 ins?)* (p.Phe216fs)	ND	Neg	DP	Neg
17	F/67	LOT	N/A	Neg	ND	Neg	DP	FP
18	M/62	LOT	N/A	ND	ND	Neg	DP	FP
19	M/69	EVT	N/A	ND	ND	Neg	DP	DP
20	F/63	EVT	N/A	ND	ND	ND	Neg	DP
21	M/34	Epithelioid AML	TSC	*TSC2* exons 1–41 deletion	ND	ND	DP	DP
22	M/61	AML	TSC	ND	ND	ND	DP	DP
23	F/67	AML	N/A	ND	ND	ND	DP	DP
24	F/37	Epithelioid AML	N/A	ND	ND	+	DP	FP
25	F/60	AML	N/A	ND	ND	Neg	DP	DP
26	F/67	Lipid-rich AML	N/A	ND	ND	ND	FP	FP

*F* female; *M* male; *N/A* not applicable; *ND* not done; *DP* diffuse positive; *FP* focal positive; *Neg* negative

### Immunohistochemistry analysis

Automated IHC was performed on formalin-fixed, paraffin-embedded whole-tissue sections of 5-μm thickness and sections of a human RCC tissue microarray (TMA) block previously constructed at the University of Wisconsin-Madison, using the Ventana Discovery Ultra BioMarker Platform (Ventana Medical Systems, Oro Valley, AZ). In brief, tissue sections were subjected to deparaffinization and heat-induced antigen retrieval with Cell Conditioner 1 (Ventana #950–224) at 95 °C for 64 min. Tissue sections were then incubated with primary antibodies against GPR143 (rabbit polyclonal antibody, #PA5-51984, 1:100; Invitrogen, Waltham, MA) or GPNMB (E4D7P rabbit monoclonal antibody, #38313, 1;100; Cell Signaling Technology, Danvers, MA) at 37 °C for 60 min. After rinsing with reaction buffer (Ventana #950–300), sections were incubated with Discovery OmniMap anti-rabbit horseradish peroxidase conjugated secondary antibody (Ventana #760–4311) for 16 min at 37 °C. Ventana UltraView DAB reagents were applied for detection, visualization and counterstaining. The sections were dehydrated and cover-slipped with permanent media.

IHC results were assessed by two pathologists and categorized as follows: diffuse positive (moderate or strong staining intensity in ≥ 80% of tumor cells), focal positive (moderate or strong staining intensity in 10—79% of tumor cells), or negative (< 10% of tumor cells staining). Histiocytes/macrophages were used as the internal positive control for GPNMB, as they showed high expression of GPNMB.

## Results

### RCCs with high expression of GPR143 mRNA were enriched for renal neoplasms with *FLCN/TSC/mTOR-TFE* alterations

Previous studies demonstrated that GPR143 is not only highly expressed in melanoma, but also is significantly upregulated in various human carcinomas, including breast cancer, colon cancer and RCC [18, 22]. By interrogating the HPA database, we found *GPR143* mRNA was expressed at high levels in the TCGA-KICH cohort of chRCC (Median: 21.5 protein-coding transcripts per million [pTPM]) and the TCGA-KIRP cohort of pRCC (Median: 12.6 pTPM) (Supplementary Fig. 1), which were ranked as the 2nd and 3rd tumor with high GPR143 expression, respectively, among 31 cohorts of 21 cancer types and just below cutaneous melanoma (Median: 151.4 pTPM).

More interestingly, although the median level of *GPR143* mRNA expression was relatively low (2.3 pTPM) in the TCGA-KIRC cohort of ccRCC, 8 of 12 (66.7%) outlier tumors with high (> 60 pTPM) *GPR143* expression in this TCGA cohort harbored genetic alterations in *FLCN/TSC/mTOR-TFE* pathway (Supplementary file 1). Similarly, 15 of 38 (39.5%) tumors with high (> 60 pTPM) *GPR143* expression in the TCGA-KIRP cohort also contained genetic changes in *FLCN/TSC/mTOR-TFE* pathway (Supplementary file 2). Among these 23 RCCs with high *GPR143* mRNA expression and *FLCN/TSC/mTOR-TFE* pathway aberrations, 13 tumors harbored *TFE3* gene rearrangement with various fusion partners, two tumors harbored *TFEB* gene fusion, and two tumors with *TFEB* amplification, indicating that these 17 tumors should be re-classified as tRCC. The other six tumors with high expression of GPR143 in the TCGA-KIRC and TCGA-KIRP cohorts harbored various mutations in *FLCN/TSC* pathway, including two tumors with *FLCN* deletion, three tumors with *TSC2* mutation and one tumor containing *TSC1* mutation, respectively (Supplementary file 1 and 2).

In contrast, although the average expression level of *GPR143* was higher (30.4 pTPM) in the TCGA-KICH cohort of chRCC when compared to the TCGA-KIRC cohort (12.7 pTPM), no *TFE3/TFEB* amplification, fusion or mutation in *FLCN/TSC1/TSC2* was observed among 6 tumors with the highest (> 60 pTPM) *GPR143* mRNA expression. Only 2 of 26 (7.7%) tumors with relatively high (> 30 pTPM) *GPR143* expression displayed *TSC1* mutation (Supplementary file 3). These results indicate many chRCC tumors intrinsically express certain amount of *GPR143* mRNA in the absence of *FLCN/TSC/mTOR-TFE* alterations.

### Renal neoplasms with *FLCN/TSC/mTOR-TFE* alterations frequently expressed high levels of GPR143 protein

**Fig. 1:**
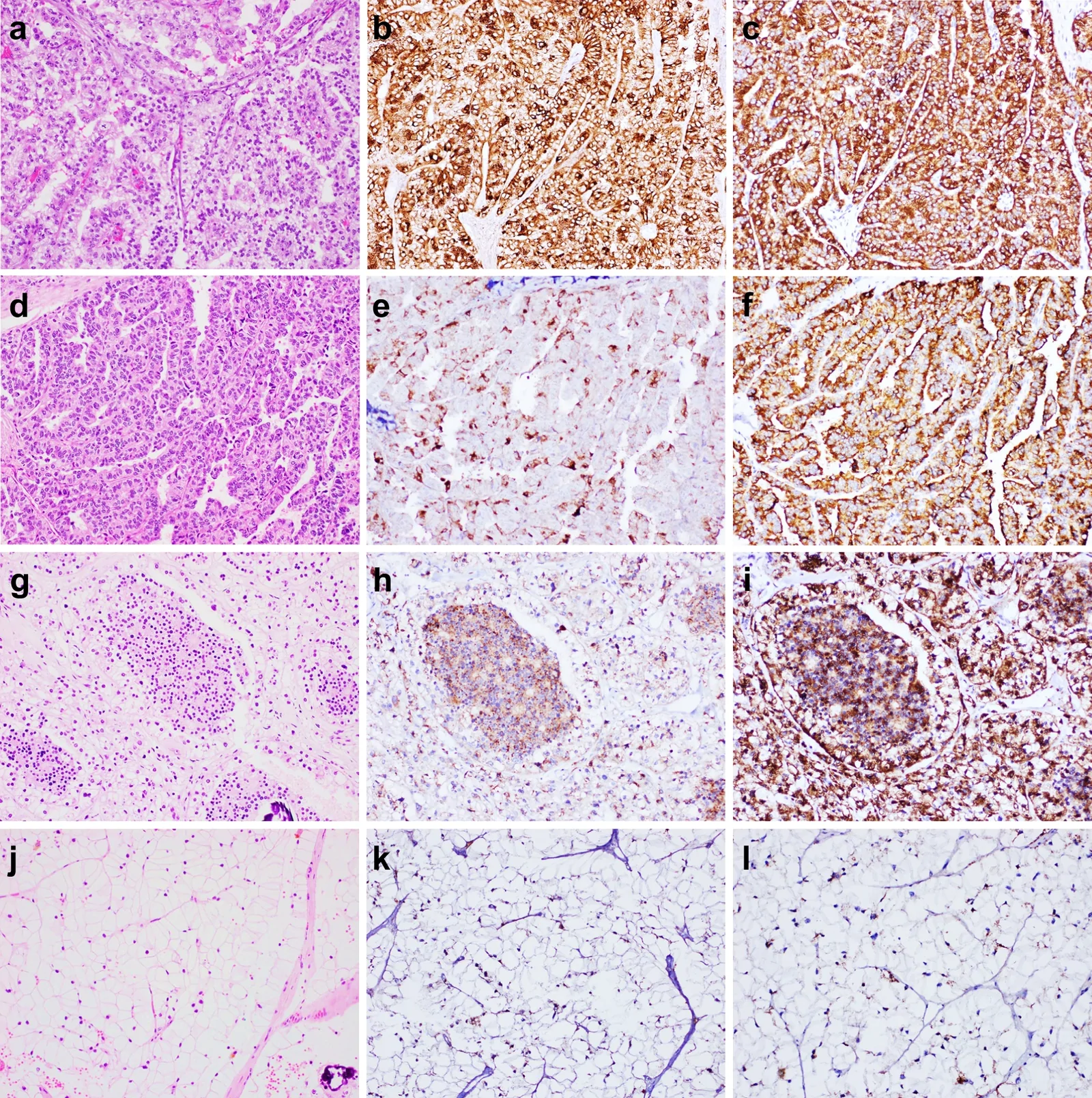
Representative H&E and IHC analysis in *TFE3*-rearranged RCCs. (**a**-**c**) Diffuse positive GPNMB (**b**) and GPR143 immunoreactivity (**c**) in a *TFE3*-rearranged RCC. (**d**-**f**) Focal GPNMB staining (**e**) and diffuse GPR143 immunoreactivity (**f**) in a FISH-confirmed *TFE3*-rearranged RCC. (**g**-**i**) Focal GPNMB staining (**h**) and diffuse GPR143 immunoreactivity (**i**) in a FISH-confirmed *TFE3*-rearranged RCC with biphasic morphology. (**j**-**l**) Negative GPNMB (**k**) and GPR143 immunoreactivity (**l**) in a FISH-confirmed *TFE3*-rearranged RCC with abundant clear cytoplasm. Original magnification: 200 ×

**Fig. 2:**
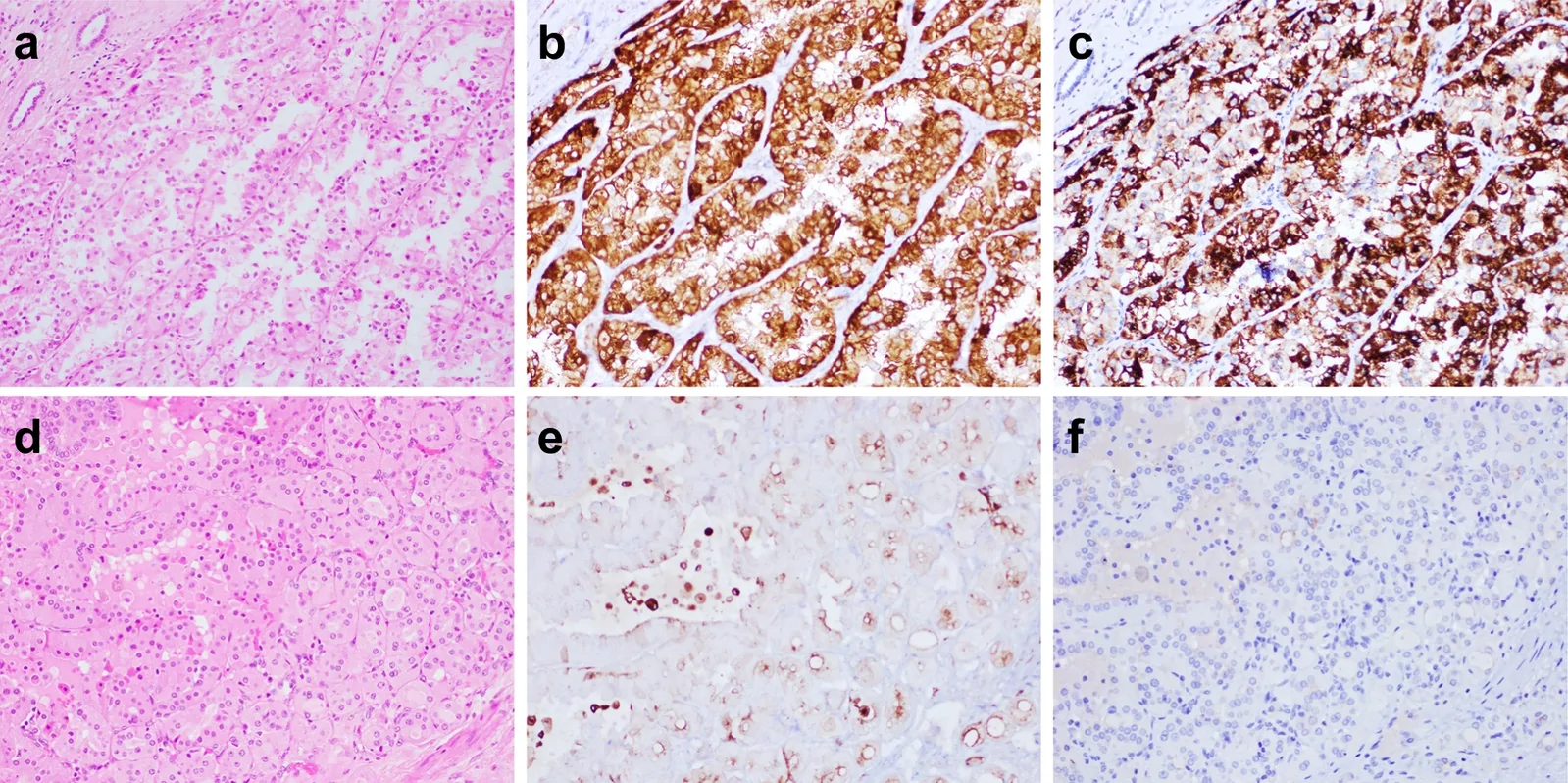
Representative H&E and IHC analysis in *TFEB*-altered RCCs. (**a**-**c**) Diffuse positive GPNMB (**b**) and GPR143 immunoreactivity (**c**) in a *TFEB*-rearranged RCC. (**d**-**f**) Focal weak GPNMB staining (**e**) and negative GPR143 immunoreactivity (**f**) in a *TFEB*-amplified RCC. Original magnification: 200 ×

To confirm the findings of upregulated expression of GPR143 in renal neoplasms with *FLCN/TSC/mTOR-TFE* alterations, IHC analysis was performed in 27 renal tumors using an antibody against GPR143. Diffuse strong GPR143 immunoreactivity was observed in eight of ten (80%) tRCCs, while diffuse GPNMB staining was detected in six of ten (60%) tRCCs (Figs. 1, 2 and Table 1). One FISH-confirmed *TFE3*-rearranged RCC with abundant clear cytoplasm showed negative for both GPR143 and GPNMB stains (Fig. 1j-l). Whereas one FISH-confirmed *TFEB*-rearranged RCC showed diffuse strong positive GPR143 and GPNMB labeling (Fig. 2a-c), one *TFEB* amplified RCC only exhibited focal patchy weak positive for GPNMB stain and negative GPR143 immunoreactivity (Fig. 2d-f). It is important to note that although most (Eight of ten) tRCCs displayed concordant expression pattern between GPR143 and GPNMB, two FISH-confirmed *TFE3*-rearranged RCCs showed diffuse positive for GPR143 and only focal scattered immunoreactivity for GPNMB (Fig. 1d-i). Interestingly, in the FISH-confirmed *TFE3*-rearranged RCC with biphasic morphology composed of large clear tumor cells and small nested eosinophilic cells clustered in the center, the immunoreactivity for GPNMB was located predominantly in the central pseudorosette structures while the large tumor cells with abundant clear cytoplasm were mainly negative for GPNMB (Fig. 1h). In contrast, both populations of tumor cells were positive for GPR143 labeling (Fig. 1i).

**Fig. 3:**
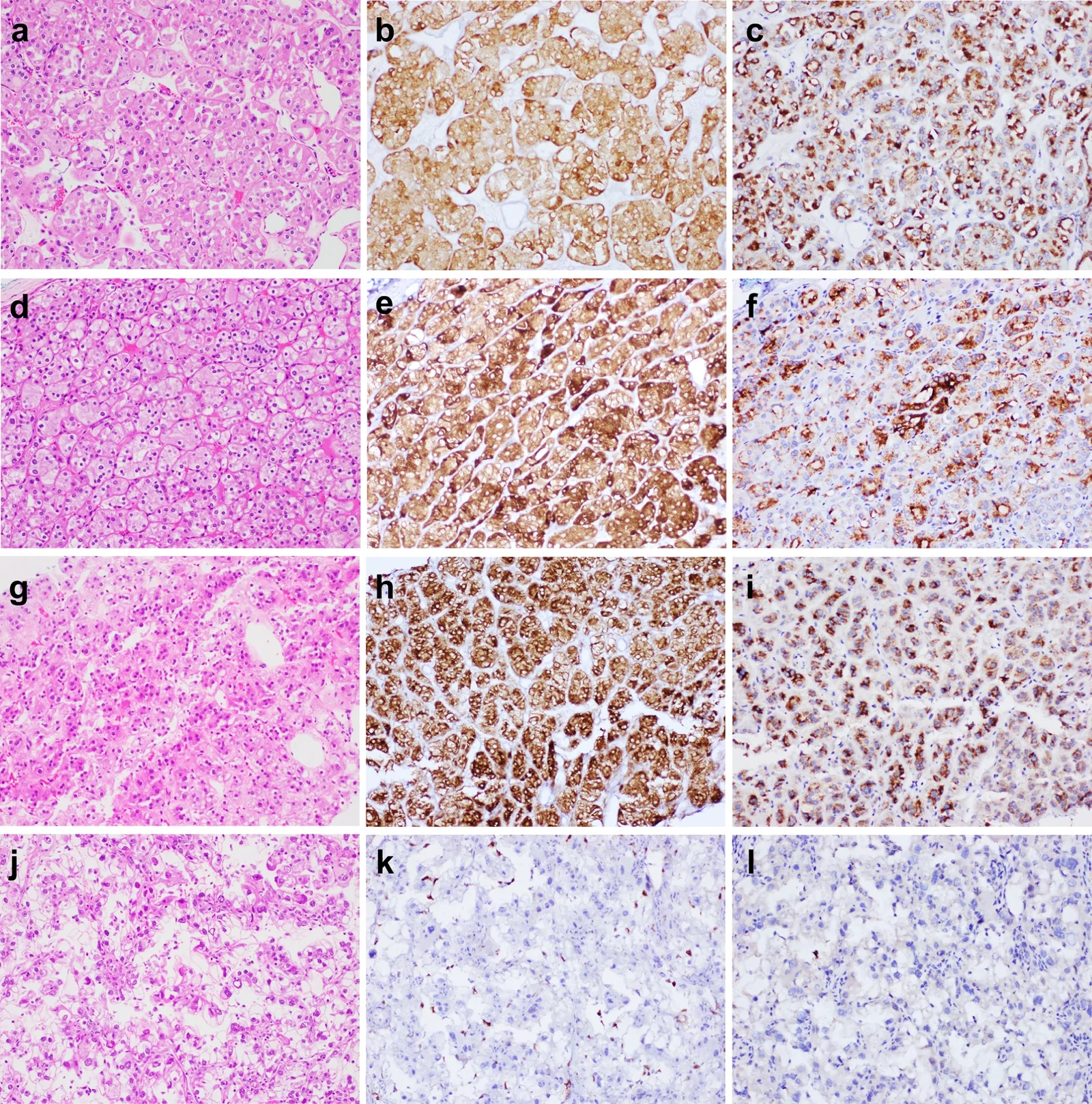
Representative H&E and IHC analysis in *FLCN* mutated renal tumors from three patients with BHD syndrome. (**a**-**f**) Diffuse positive GPNMB (b and e) and GPR143 immunoreactivity (**c** and **f**) in two HOCTs from one BHD patient with a germline *FLCN* mutation. (**g**-**i**) Diffuse positive GPNMB (**h**) and GPR143 immunoreactivity (**i**) in an unclassified low grade oncocytic renal neoplasm from one BHD patient with a germline *FLCN* mutation. (**j**-**l**) Negative GPNMB (**k**) and GPR143 immunoreactivity (**l**) in a ccRCC from one BHD patient with a germline *FLCN* mutation. Original magnification: 200 ×

**Fig. 4:**
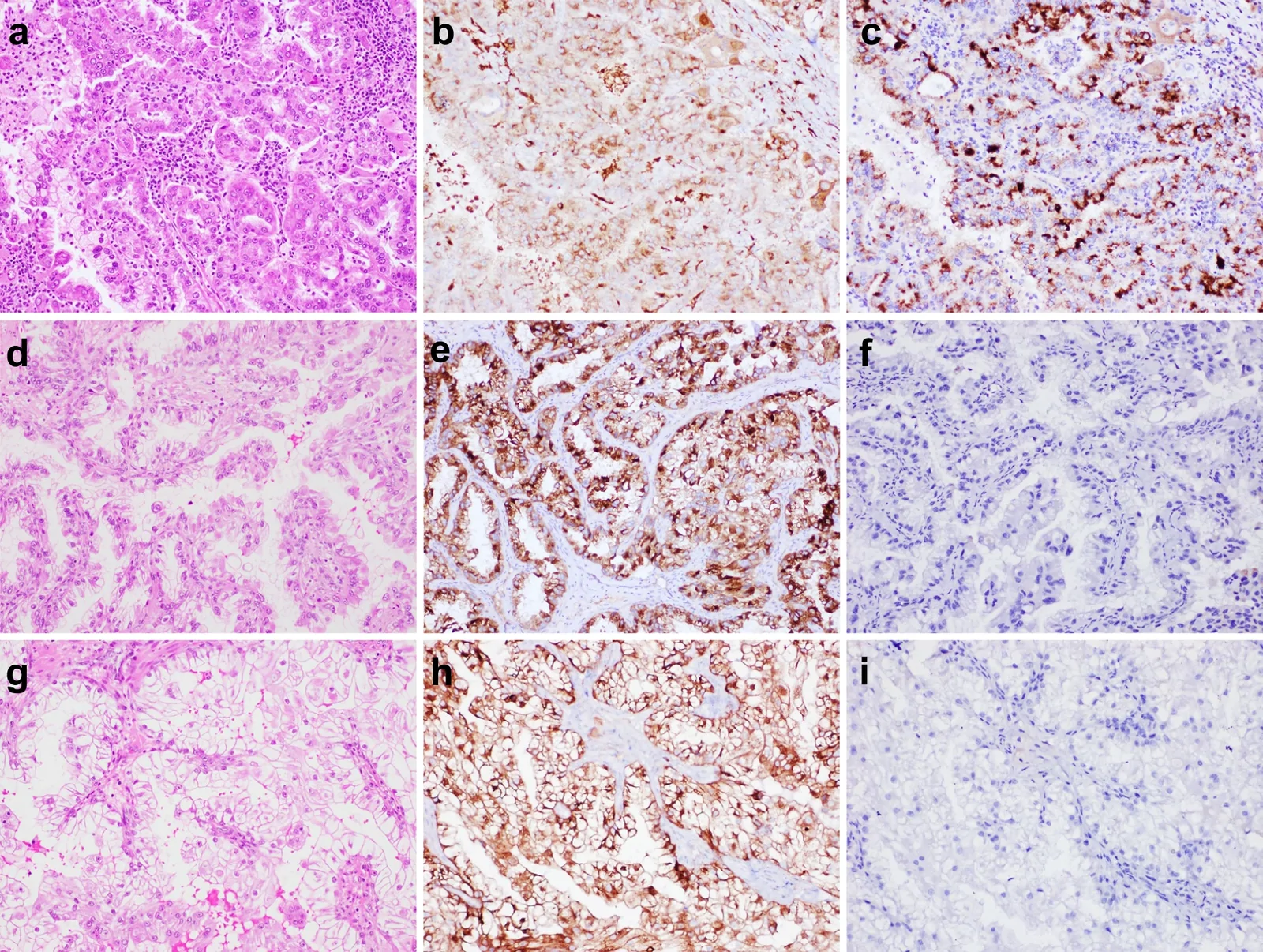
Representative H&E and IHC analysis in *TSC1* mutated renal tumors. (**a**-**c**) Diffuse moderate GPNMB staining (**b**) and diffuse positive GPR143 immunoreactivity (**c**) in one ESC RCC with a germline *TSC1* mutation. (**d**-**i**) Diffuse positive GPNMB staining (**e** and **h**) and negative GPR143 immunoreactivity (**f** and **i**) in two unclassified *TSC1* mutated RCCs from two patients with TSC and a germline *TSC1* mutation. Original magnification: 200 ×

**Fig. 5:**
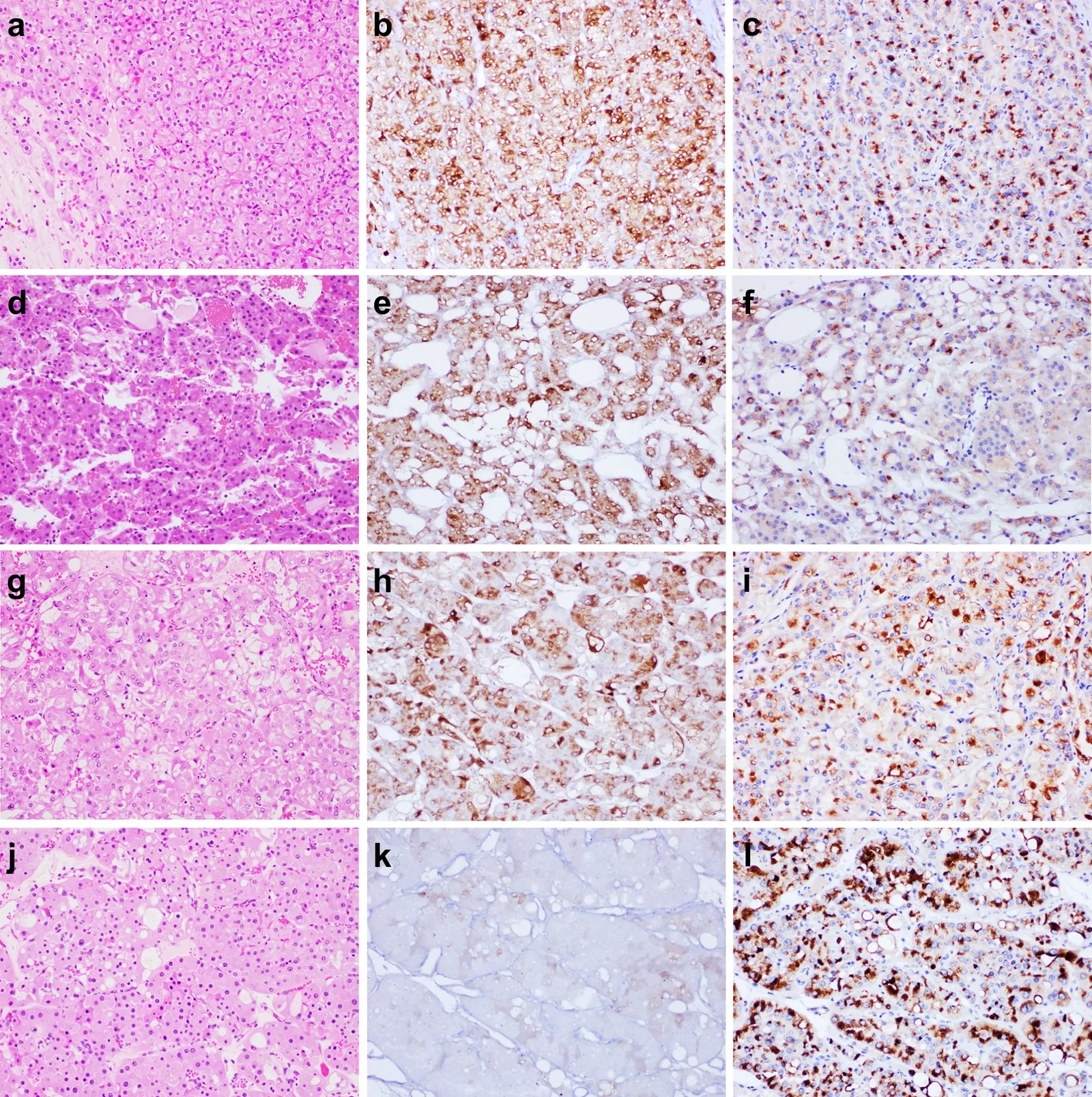
Representative H&E and IHC analysis in oncocytic renal neoplasms. (**a**-**f**) Diffuse positive GPNMB (**b** and **e**) and focal GPR143 immunoreactivity (c and f) in two cases of LOT. (**g**-**i**) Diffuse positive GPNMB (h) and GPR143 immunoreactivity (**i**) in one EVT case. (**j**-**l**) Negative GPNMB (**k**) and diffuse positive GPR143 immunoreactivity (**l**) in one EVT case. Original magnification: 200 ×

**Fig. 6:**
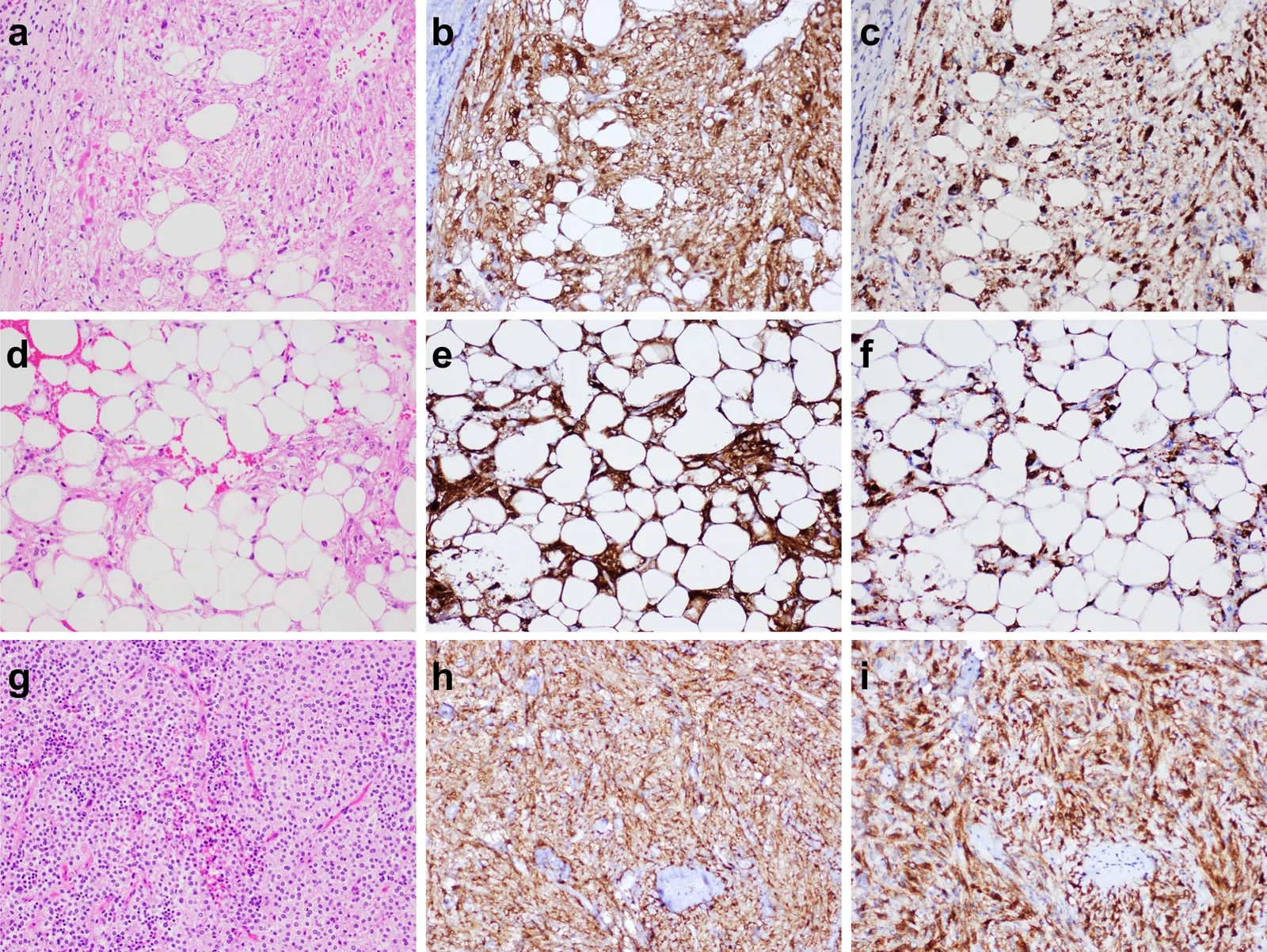
Representative H&E and IHC analysis in renal angiomyolipomas (AMLs). (**a**-**c**) Diffuse positive GPNMB (**b**) and GPR143 immunoreactivity (**c**) in a renal AML from a patient with TSC. (**d**-**f**) Positive GPNMB (**e**) and GPR143 immunoreactivity (**f**) in a lipid-rich AML. (**g**-**i**) Positive GPNMB (**h**) and GPR143 immunoreactivity (**i**) in a renal epithelioid AML. Original magnification: 200 ×

In four *FLCN*-mutated renal tumors from three BHD syndrome patients with germline *FLCN* mutations, GPR143 and GPNMB immunostains were diffusely positive in three oncocytic tumors, but negative in a ccRCC with a germline *FLCN c.1285dup* (p.His429fs) mutation (Fig. 3). Diffuse GPNMB labeling was also found in one of one (100%) ESC with a germline *TSC1* mutation (Fig. 4b), two of two (100%) LOT and one of two (50%) EVT cases (Fig. 5), while focal or diffuse GPR143 staining was observed in these five oncocytic tumors (Figs. 4c and 5). Interestingly, in comparison to strong granular cytoplasmic and cell membrane staining in tRCCs, GPR143 stain in most of these oncocytic tumors exhibited fine granular or dot-like patterns, which may be attributed to its localization in intracellular organelles. In contrast, two *TSC1* mutated RCC exhibited diffuse strong positive for GPNMB, but negative for GPR143 labeling (Fig. 4d-i). Furthermore, all six renal AMLs tested were positive for both GPR143 and GPNMB immunostains (Fig. 6 and Table 1). Taken together, these data demonstrated that overlapping expression of GPR143 and GPNMB were observed in most but not all of renal neoplasms with *FLCN/TSC/mTOR-TFE* alterations, and GPR143 immunostain was positive in some cases with negative or focal GPNMB labeling.

To evaluate the specificity of GPR143 stain for renal neoplasms with *FLCN/TSC/mTOR-TFE* alterations, GPR143 and GPNMB IHC analyses were performed on a human RCC TMA, which consists of a large number of ccRCC, pRCC, chRCC and oncocytoma. We found that 1 of 91 (1.1%) ccRCCs showed strong positive for GPNMB and GPR143 stains, 6 of 91 (6.6%) ccRCCs displayed weak GPNMB stain with negative GPR143 labeling. For pRCC, 2 of 45 (4.4%) tumors showed positive for both GPNMB and GPR143 stains, 6 of 45 (13.3%) tumors displayed positive GPNMB immunoreactivity with negative GPR143 labeling. No ccRCC or pRCC demonstrated GPNMB-GPR143 + immunostain pattern (Supplemental Table 1). Among 15 chRCCs tested on the tissue microarray, one tumor exhibited GPNMB-GPR143 + stain pattern and 3 tumors showed positive GPNMB immunoreactivity with negative GPR143 labeling. For oncocytoma, 3 of 47 (6.4%) tumors showed positive for both GPNMB and GPR143 stains, 4 of 47 (8.5%) tumors displayed positive GPNMB immunoreactivity with negative GPR143 labeling (Supplemental Table 1). Independently, we also examine the GPR143 immunoreactivity on regular resection slides of three most common histological types of RCC. Only 1 of 17 (5.9%) ccRCC showed focal moderate GPR143 stain in < 30% tumor area. One of seven (14.3%) pRCC cases exhibited diffuse strong immunoreactivity for both GPR143 and GPNMB, and negative for TFE3, HMB45 and MART-1 stains (Supplementary Fig. 2 and data not shown). Two of eight (25%) chRCC tested displayed diffuse positive for GPR143, while five of eight (62.5%) chRCC showed diffuse GPNMB immunoreactivity (Supplementary Fig. 3). Taken together, these results suggest neither GPR143 nor GPNMB are specifically expressed in tRCCs or *FLCN/TSC* mutated renal neoplasms, and a significant fraction of chRCC and a small subset of oncocytoma could display positivity for these two biomarkers.

## Discussion

tRCC is a group of molecularly defined RCCs, consisting of *TFE3*-rearranged RCC, *TFEB*-rearranged RCC and *TFEB*-amplified RCC. The diagnosis of tRCCs mainly relies on FISH analyses, which are expensive and not easily available in most clinical laboratories. It has been shown that IHCs using several biomarkers, including TFE3, cathepsin K and GPNMB, are useful ancillary screening tests to identify tRCCs. However, the sensitivities of these biomarkers are not very high and the interpretation of ambiguous TFE3 and GPNMB immunostains could be challenging [1, 2, 5, 23]. It has been proposed that only diffuse GPNMB immunoreactivity is predictive of *FLCN/TSC/mTOR-TFE* alterations in renal tumors [1, 2, 5]. In this study, we demonstrated that a large fraction of tumors with high levels of *GPR143* expression in TCGA-KIRC and TCGA-KIRP cohorts harbored *TFE3/TFEB* alterations. Consistently, significant overexpression of *GPR143* in tRCC was found by RNA-seq analysis of 15 *TFE3*-rearranged tRCC samples as well as TFE3 ChIP-seq test using the UOK146 cell line harboring a *PRCC-TFE3* gene fusion [24]. To confirm upregulated GPR143 protein in tRCC, we performed IHC analysis with an antibody against GPR143 and demonstrated positive GPR143 immunoreactivity in the majority of tRCC cases tested. Moreover, two FISH-confirmed *TFE3*-rearranged RCCs with focal scattered immunoreactivity for GPNMB showed diffuse positive for GPR143. Our results suggest that complementary to the aforementioned existing markers, GPR143 could serve as a useful adjunct marker to increase the sensitivity for tRCC screen and diagnosis. Multiplex IHC analysis using a cocktail of antibodies against these biomarkers could be an ideal diagnostic tool to efficiently screen tRCCs with a lower false negative rate and simultaneously save the limited biopsy tissues for subsequent molecular tests.

In addition, we showed that similar to GPNMB IHC analysis, GPR143 immunoreactivity was observed in the majority of *FLCN* mutated renal tumors and renal neoplasms with high frequencies of *TSC/mTOR* signal pathway aberrations, indicating that GPR143 could also function as a surrogate marker for *FLCN/TSC/mTOR* alterations in renal tumors. However, we also found that a significant fraction of chRCC cases displayed diffuse positive labeling for these two biomarkers. Although aberrant TSC/mTOR signaling pathway has been implicated in the pathogenesis and disease progression in a subset of chRCC [25, 26], additional mechanisms independent of *FLCN/TSC* mutations or mTOR pathway activation, such as loss of transcriptional repressors or epigenetic changes, could play roles in upregulation of GPR143 and GPNMB in some chRCC and other oncocytic renal tumors. Moreover, it is also possible that the granular and dot-like staining pattern of GPR143 observed in most of *FLCN/TSC/mTOR*-related oncocytic renal tumors could be partially attributed to increased mitochondria or dysfunctional lysosome and other intracellular organelles. Therefore, further studies with larger cohorts of chRCC and other oncocytic renal neoplasms with *FLCN/TSC/mTOR* alterations are warranted to evaluate the specificity of these biomarkers for *FLCN/TSC/mTOR* alteration prediction. Moreover, we found that dying tumor cells with paraptosis, normal epithelial cells in few renal proximal tubules and degenerative epithelial cells with large, vacuolated cytoplasm could show focal strong GPR143 immunoreactivity, possibly due to increased lysosomal activity. Hence, these nonspecific stainings with GPR143 antibody should not be interpretated as true positive immunoreactivity.

In this study, we showed that both GPNMB and GPR143 immunostains yielded negative results in one FISH-confirmed *TFE3*-rearranged RCC with abundant clear cytoplasm. These two biomarkers were also negative in one ccRCC with a germline *FLCN* frameshift mutation. Consistently, a recent study described positive GPNMB immunoreactivity in only 25% (two of eight) of ccRCC cases with coexisting *VHL* and *TSC*/*mTOR* mutations [9]. Taken together, these results indicate that relative to the high predictive value in oncocytic renal neoplasms, both GPNMB and GPR143 immunoreactivity may be insensitive indicators for *FLCN/TSC/mTOR*-*TFE* aberrations in renal tumors with clear cytoplasm. The underlying mechanisms for lower positive rates of GPNMB and GPR143 stains in these tRCCs or mTOR-activated renal tumors with clear cell morphology remain unclear, although accumulation of glycogen and lipids, altered lysosomal/organelle trafficking or increased mitochondrial autophagy could play contributary roles.

*TFEB*-altered RCCs are much less common than *TFE3*-rearranged RCCs. Among *TFEB*-altered RCCs, the incidence of *TFEB-*amplified RCC seems to be relatively lower compared to that of *TFEB*-rearranged RCC. Currently, approximately 100 cases of *TFEB-*amplified RCC have been reported in the literature [27–29]. It has been shown in recent studies that negative or patchy/equivocal GPNMB immunoreactivity was observed in 36.4%—50% of *TFEB-*amplified RCCs [2, 30], which indicates the sensitivity of GPNMB stain in *TFEB-*amplified RCCs could be significantly lower than that in *TFE3* or *TFEB*-rearranged RCCs. Due to the small cohort of our study and the rare incidence of *TFEB-*altered RCCs, we only included a *TFEB*-rearranged RCC case, which exhibited diffuse GPNMB and GPR143 labeling. In contrast, the *TFEB-*amplified RCC case in this study showed focal patchy GPNMB staining and negative for GPR143 labeling, indicating that GPR143 may not be superior to GPNMB for identification of *TFEB* amplified RCCs. However, given that two *TFEB-*amplified RCC cases in the TCGA cohorts expressed high levels of *GPR143* mRNA (Supplementary file 1 and 2), it is still vey likely that a significant subset of, if not the majority of, *TFEB-*amplified RCCs could display positive GPR143 immunoreactivity. Therefore, further study is needed to compare the staining patterns and positive rates of GPNMB and GPR143 immunostains in this specific subtype of *TFEB*-altered RCCs.

In addition to the low number of tRCC cases and *FLCN/TSC/mTOR*-related renal tumors, the other limitations of our study include the retrospective nature of study from a single tertiary care center and the lack of molecular confirmation of *TSC/mTOR-TFE* aberrations in a significant proportion of this cohort*.* However, given that GPR143 immunostain was positive in several renal tumors with negative or focal GPNMB staining, we believe that our findings of GPR143 expression in renal neoplasms with *FLCN/TSC/mTOR-TFE* alterations should be of clinical significance. These results not only provide a novel adjunct marker for precise diagnosis of renal tumors with overlapping morphology, but also have potential values for subclassification and management of kidney cancer patients based on the aberrantly activated signal pathways. Interestingly, in a recent study, epigenomic profiling of circulating nucleosomes in plasma samples from patients with tRCC using cell-free ChIP-seq technology revealed increased H3K4me3 and H3K27ac signals at the *GPR143* gene locus were detected specifically in tRCC plasma. The result further confirmed GPR143 is highly selective marker for tRCC and suggested that TFE3-driven epigenomic signature in plasma could be used as a noninvasive tool for detection, diagnosis and monitoring of tRCC [31]. Due to the high frequencies of positive GPR143 immunoreactivity in *TFE3/TFEB*-rearranged RCC and renal AML, it will be interesting to study GPR143 expression in other *TFE*-altered conditions, such as alveolar soft part sarcomas and non-renal perivascular epithelioid cell tumor. Since GPR143 is a MiT/TFE family of transcriptional target implicated in melanosome biogenesis and maturation [14–16], the potential clinical application of GPR143 immunostain could be extended to several cutaneous and soft tissue disorders upon optimization and validation, which could provide another useful melanocytic differentiation marker to facilitate the diagnosis of various melanocytic lesions and clear cell sarcoma of soft tissue.

In summary, by interrogating the HPA and cBioPortal databases, high frequencies of *FLCN/TSC/mTOR-TFE* alterations were identified among RCCs with high levels of GPR143 expression in two TCGA kidney cancer cohorts. Similar to GPNMB labeling, GPR143 immunostain was positive in the majority of tRCC cases and renal tumors with *FLCN/TSC/mTOR* alterations, suggesting that GPR143 could function as another surrogate marker for *FLCN*/*TSC/mTOR-TFE* signal pathway aberrations in certain renal tumors. Despite the overlapping expression of GPR143 and GPNMB in most renal neoplasms with *FLCN/TSC/mTOR-TFE* alterations, diffuse GPR143 immunoreactivity was found in some cases with negative or focal GPNMB staining. These results indicate GPR143 could serve as a useful adjunct marker to improve the sensitivity for screening renal tumors with *FLCN/TSC/mTOR-TFE* alterations.

## Supplementary Information

Below is the link to the electronic supplementary material.

Supplementary file1 (JPG 203 KB)

Supplementary file2 (JPG 977 KB)

Supplementary file3 (JPG 1322 KB)

Supplementary file4 (XLSX 10 KB)

Supplementary file5 (XLSX 12 KB)

Supplementary file6 (XLSX 11 KB)

Supplementary file7 (DOCX 21 KB)

## References

[1] Salles DC, Asrani K, Woo J et al (2022) GPNMB expression identifies TSC1/2/mTOR-associated and MiT family translocation-driven renal neoplasms. J Pathol 257(2):158–171. 10.1002/path.5875 35072947 PMC9310781

[2] Li H, Matoso A (2025) Unlocking diagnostic potential: a retrospective analysis of GPNMB immunohistochemistry in nearly 1000 surgical pathology specimens. Hum Pathol 159:105799. 10.1016/j.humpath.2025.105799 40389121

[3] Whaley RD, Sill DR, Tekin B et al (2025) Evaluation of 3,606 renal cell tumors for TFE3 rearrangements and TFEB alterations via fluorescence in situ hybridization, next generation sequencing, and GPNMB immunohistochemistry. Hum Pathol 159:105797. 10.1016/j.humpath.2025.105797 40381702

[4] Furuya M, Hong SB, Tanaka R et al (2015) Distinctive expression patterns of glycoprotein non-metastatic B and folliculin in renal tumors in patients with Birt-Hogg-Dube syndrome. Cancer Sci 106(3):315–323. 10.1111/cas.12601 25594584 PMC4376441

[5] Argani P, Baraban E, Yaskiv O et al (2025) Clinically sporadic folliculin -mutated renal epithelial neoplasms represent a mixture of true somatic folliculin -mutated and occult Birt-Hogg-Dube syndrome-associated cases : morphologic and molecular overlap with TSC/MTOR -mutated eosinophilic renal neoplasms and MiT family translocation renal cell carcinoma. Am J Surg Pathol 49(9):859–872. 10.1097/PAS.0000000000002413 40321032

[6] Xia QY, Wang XT, Zhang HZ et al (2025) GPNMB expression identifies FLCN-associated eosinophilic renal tumors with heterogeneous clinicopathologic spectrum and gene expression profiles. Am J Surg Pathol. 10.1097/PAS.0000000000002435 40470587

[7] Li H, Argani P, Halper-Stromberg E et al (2023) Positive GPNMB immunostaining differentiates renal cell carcinoma with fibromyomatous stroma associated with TSC1/2/MTOR alterations from others. Am J Surg Pathol 47(11):1267–1273. 10.1097/PAS.0000000000002117 37661807 PMC10592185

[8] Wu Y, Zhang H, Liu Y et al (2025) Comprehensive analysis of 15 cases of ELOC-RCC and identification of novel mutation site. Am J Surg Pathol. 10.1097/PAS.0000000000002443 40557827

[9] Chen M, Liu Y, Wu Y et al (2025) Evaluation of nuclear pseunclusions, GATA3, 34betaE12, and GPNMB performances in CK7 diffuse positive clear cell renal cell neoplasms. Hum Pathol. 165:105938. 10.1016/j.humpath.2025.105938 40975181

[10] Bjornsson J, Short MP, Kwiatkowski DJ, Henske EP (1996) Tuberous sclerosis-associated renal cell carcinoma. Clinical, pathological, and genetic features. Am J Pathol. 149(4):1201–8 8863669 PMC1865172

[11] Camparo P, Vasiliu V, Molinie V et al (2008) Renal translocation carcinomas: clinicopathologic, immunohistochemical, and gene expression profiling analysis of 31 cases with a review of the literature. Am J Surg Pathol 32(5):656–670. 10.1097/PAS.0b013e3181609914 18344867

[12] Martignoni G, Pea M, Gobbo S et al (2009) Cathepsin-K immunoreactivity distinguishes MiTF/TFE family renal translocation carcinomas from other renal carcinomas. Mod Pathol 22(8):1016–1022. 10.1038/modpathol.2009.58 19396149

[13] Wang XM, Zhang Y, Mannan R et al (2021) TRIM63 is a sensitive and specific biomarker for MiT family aberration-associated renal cell carcinoma. Mod Pathol 34(8):1596–1607. 10.1038/s41379-021-00803-z 33854184 PMC8298271

[14] Bassi MT, Schiaffino MV, Renieri A et al (1995) Cloning of the gene for ocular albinism type 1 from the distal short arm of the X chromosome. Nat Genet 10(1):13–19. 10.1038/ng0595-13 7647783

[15] Schiaffino MV, Baschirotto C, Pellegrini G et al (1996) The ocular albinism type 1 gene product is a membrane glycoprotein localized to melanosomes. Proc Natl Acad Sci U S A 93(17):9055–9060. 10.1073/pnas.93.17.9055 8799153 PMC38594

[16] Vetrini F, Auricchio A, Du J et al (2004) The microphthalmia transcription factor (Mitf) controls expression of the ocular albinism type 1 gene: link between melanin synthesis and melanosome biogenesis. Mol Cell Biol 24(15):6550–6559. 10.1128/MCB.24.15.6550-6559.2004 15254223 PMC444869

[17] Giordano F, Simoes S, Raposo G (2011) The ocular albinism type 1 (OA1) GPCR is ubiquitinated and its traffic requires endosomal sorting complex responsible for transport (ESCRT) function. Proc Natl Acad Sci U S A 108(29):11906–11911. 10.1073/pnas.1103381108 21730137 PMC3141971

[18] Lee YJ, Shin KJ, Jang HJ et al (2023) GPR143 controls ESCRT-dependent exosome biogenesis and promotes cancer metastasis. Dev Cell 58(4):320-334 e8. 10.1016/j.devcel.2023.01.006 36800996

[19] Baba M, Furuya M, Motoshima T et al (2019) TFE3 Xp11.2 translocation renal cell carcinoma mouse model reveals novel therapeutic targets and identifies GPNMB as a diagnostic marker for human disease. Mol Cancer Res 17(8):1613–1626. 10.1158/1541-7786.MCR-18-1235 31043488 PMC6679785

[20] Uhlen M, Zhang C, Lee S et al (2017) A pathology atlas of the human cancer transcriptome. Science. 10.1126/science.aan2507 28818916

[21] Cerami E, Gao J, Dogrusoz U et al (2012) The cBio cancer genomics portal: an open platform for exploring multidimensional cancer genomics data. Cancer Discov 2(5):401–404. 10.1158/2159-8290.CD-12-0095 22588877 PMC3956037

[22] Bai R, Yin P, Xing Z et al (2024) Investigation of GPR143 as a promising novel marker for the progression of skin cutaneous melanoma through bioinformatic analyses and cell experiments. Apoptosis 29(3–4):372–392. 10.1007/s10495-023-01913-6 37945816

[23] Sharain RF, Gown AM, Greipp PT, Folpe AL (2019) Immunohistochemistry for TFE3 lacks specificity and sensitivity in the diagnosis of TFE3-rearranged neoplasms: a comparative, 2-laboratory study. Hum Pathol 87:65–74. 10.1016/j.humpath.2019.02.008 30851332

[24] Lee CR, Suh J, Jang D et al (2024) Comprehensive molecular characterization of TFE3-rearranged renal cell carcinoma. Exp Mol Med 56(8):1807–1815. 10.1038/s12276-024-01291-2 39085357 PMC11372160

[25] Davis CF, Ricketts CJ, Wang M et al (2014) The somatic genomic landscape of chromophobe renal cell carcinoma. Cancer Cell 26(3):319–330. 10.1016/j.ccr.2014.07.014 25155756 PMC4160352

[26] Roldan-Romero JM, Santos M, Lanillos J et al (2020) Molecular characterization of chromophobe renal cell carcinoma reveals mTOR pathway alterations in patients with poor outcome. Mod Pathol 33(12):2580–2590. 10.1038/s41379-020-0607-z 32616874

[27] Kuroda N, Sugawara E, Ohe C et al (2021) Review of TFEB-amplified renal cell carcinoma with focus on clinical and pathobiological aspects. Pol J Pathol 72(3):197–199. 10.5114/pjp.2021.111769 35048631

[28] Lobo J, Rechsteiner M, Helmchen BM, Rupp NJ, Weber A, Moch H (2022) Eosinophilic solid and cystic renal cell carcinoma and renal cell carcinomas with TFEB alterations: a comparative study. Histopathology 81(1):32–43. 10.1111/his.14663 35403742

[29] Zhang M, Xian J, Tang J et al (2026) Clinicopathologic and molecular study of TFEB -altered renal cell carcinomas: tumors with frequent PDL1 expression. Am J Surg Pathol 50(1):84–102. 10.1097/PAS.0000000000002458 40704447

[30] Pivovarcikova K, Grossmann P, Steiner P et al (2026) Immunohistochemical expression of GPNMB in TFEB-rearranged and TFEB-amplified renal cell carcinomas. Virchows Arch. 10.1007/s00428-026-04466-6 41779042 PMC13582045

[31] Garinet S, Semaan K, Li J et al (2026) Cell-free DNA epigenomic profiling enables noninvasive detection and monitoring of translocation renal cell carcinoma. J Clin Invest. 10.1172/JCI195725 41623182 PMC12867156

