# Supplementary material for: GPR143, a novel immunohistochemical marker for renal tumors with FLCN/TSC/MTOR-TFE alterations
Source: Virchows Arch. 2026 Jul 17;489(3):619–30. doi: 10.1007/s00428-026-04646-4 (PMC13582269; doi:10.1007/s00428-026-04646-4)
Supplement: Supplementary file 1 [file 428_2026_4646_MOESM7_ESM.docx]

| **Supplementary table 1. GPNMB and GPR143 IHC analyses of common renal tumors in a tissue microarray** | | | | |
| --- | --- | --- | --- | --- |
| Renal tumor type | GPNMB+  GPR143+ tumor, n (%) | GPNMB-GPR143+ tumor, n (%) | GPNMB+ GPR143- tumor, n (%) | GPNMB-GPR143- tumor, n (%) |
| ccRCC (n = 91) | 1 (1.1) | 0 (0) | 6 (6.6) | 84 (92.3) |
| pRCC (n = 45) | 2 (4.4) | 0 (0) | 6 (13.3) | 37 (82.2) |
| chRCC (n = 15) | 0 (0) | 1 (6.7) | 3 (20) | 11 (73.3) |
| Oncocytoma (n = 47) | 3 (6.4) | 0 (0) | 4 (8.5) | 40 (85.1) |
